# Open Surgery for Abdominal Aortic Aneurysm: 980 Consecutive Patient Outcomes from a High-Volume Centre in the United Kingdom

**DOI:** 10.1177/15385744221149585

**Published:** 2023-01-30

**Authors:** Robert Blair, Denis Harkin, Dorothy Johnston, Adrian Lim, Lisa McFetridge, Hannah Mitchell

**Affiliations:** 1 Belfast Vascular Centre, Royal Victoria Hospital, Belfast Health and Social Care Trust, Belfast, UK; 2 Royal College of Surgeons in Ireland University of Medicine and Health Sciences, Dublin, Ireland; 3 Mathematical Sciences Research Centre, 227990 Queen’s University Belfast, Belfast, UK

**Keywords:** abdominal aortic aneurysm, AAA, ruptured abdominal aortic aneurysm, open AAA repair, in-hospital mortality, risk factors, surgical repair, survival

## Abstract

**Background:**

Controversy persists regarding the optimal treatment for large abdominal aortic aneurysm (AAA), highlighted by the publication of the National Institute for Health and Care Excellence (NICE) guideline (NG156) on March 2020. The pendulum of opinion swings once more from endovascular to open surgical treatment. We report our experience over the last 15 years in treating consecutive AAA by open surgery.

**Methods:**

A retrospective review of a prospectively collected vascular database of all patients undergoing infra-renal open abdominal aortic aneurysm repair (OR) repair from 2004 to 2019 at the largest aneurysm centre in the United Kingdom. OR for elective and emergency (ruptured and symptomatic) outcomes included early morbidity and 30-day mortality, and long-term survival.

**Results:**

1017 patients underwent OR between 2004-2019, on application of our inclusion-criteria 994 patients formed our cohort for analysis (81.2% male) with a mean age 73.6 ± 7.8 years treated by OR for AAA. In that group 672 were elective and 308 were emergency (for ruptured or symptomatic). Overall 30 day mortality was 11.3%, elective 30 day mortality was 2.5%, and emergency 30 day mortality was 29.9%. 30 day re-intervention rate was 9.5%, (elective 7.0%, emergency 15.9%). Survival at 1000 days for elective repair was 72 v 46.7% for emergency and at 2000 days was 43.4% for elective v 25% for emergency.

**Conclusion:**

Our data confirm that open surgery for AAA can be performed in large volume centres quite safely. Elective and Emergency surgery does affect early 30 day mortality but does not influence long-term outcome.

## Introduction

In 1951, Dubost and colleagues reported the first successful open repair (OR) of an abdominal aortic aneurysm (AAA) and by the 1990s it had become the established treatment for large aneurysms to prevent rupture and death.^1^ By the late 1990s, 3 large Randomised Controlled Trial (RCT), EVAR-1, DREAM, OVER, demonstrated significant benefit for EVAR over OR in respect to short-term morbidity and mortality and EVAR became the first-line treatment for AAA.^2-4^ However, clinicians had become increasingly aware of mid- and long-term complications after EVAR, the publication of late follow-up from the major EVAR Trials confirmed that those early benefits from EVAR are offset by increased late complications prompting much reflection in the vascular community.^5,6^

The European Society for Vascular Surgery (ESVS) published Clinical Practice Guidelines on the Management of Abdominal Aorto-iliac Artery Aneurysms in 2019, promoting a liberal use of EVAR as the preferred treatment modality in most patients with suitable anatomy and reasonable life expectancy, but interestingly did caution that it was reasonable to consider an open surgical first strategy in younger, fit patients with a long-life expectancy of more than 10 to 15 years.^7^ On 19 March 2020, in the United Kingdom (UK) the National Institute for Health and Care Excellence (NICE) guideline on Abdominal Aortic Aneurysm: Diagnosis and Management [NG156] was published^8^ and in contrast to the majority of contemporary vascular surgery practice, and indeed the aforementioned ESVS Guidelines, unequivocally recommends open surgery as first-line elective treatment for large AAA.^7,9^ The NICE guidelines recommend Endovascular Aneurysm Repair (EVAR) should only be considered for patients with hostile abdomens, medical comorbidities or anaesthetic risks that contra-indicate open surgery. These apparently paradoxical clinical guidelines arising from expert consideration of similar clinical trial evidence raises many dilemmas for vascular surgeons, further compounded by the knowledge that outcomes from these highly selected patient-populations in clinical trials do not always translate into real-world clinical practice.

To better evaluate the likely historical and contemporary real-world outcomes, we performed a retrospective analysis of outcomes in consecutive patients who had undergone open AAA repair, elective and emergency, between 2004 and 2019. We also look at mortality, major complications, surgical reinterventions and long-term survival.^10,11^

## Methods

A retrospective review of a prospectively collected database was conducted of all open AAA repairs performed from 2004 to 2019 at the Royal Victoria Hospital Belfast (RVH), the largest aneurysm centre in the United Kingdom (UK). Incomplete data were obtained through chart review and interrogation of the Electronic Care Record (ECR). Patients with infrarenal abdominal aortic aneurysm (AAA) were included. We excluded Patients with: missing preoperative and postoperative data; thoraco-abdominal aneurysm; mycotic aneurysm; aortic dissection; aorto-enteric fistula; endovascular aneurysm repair (EVAR); secondary OR with explant of EVAR. We considered the urgency of presentation and for the purposes of the following analysis we considered patients either Elective or Emergency, which includes ruptured and symptomatic AAA patients.

These patients received open surgery in a non-randomized fashion at the discretion of the Consultant surgeon after consideration of such issues as the aneurysm anatomy and patient’s comorbidities and preference. Open repair in all cases was performed with a midline incision and transperitoneal approach, reflecting the individual surgeons preference, and the repair performed with an interposition straight or bifurcated graft (Dacron), depending on the extent of the aneurysm or any coexisting aortoiliac occlusive disease.

Patients’ medical records and laboratory results throughout the period were reviewed and demographic variables including age, sex and previous medical history (hypertension, diabetes mellitus, cardiovascular disease including coronary artery disease, heart failure and stroke) were recorded. In addition, the surgical approach, destination post procedure, level-of-care, post-operative complications and mortality at 30 days were collected along with long-term follow up data.

The project was registered with the clinical audit department of our institution (BHSCT) and additional research ethics approval was not considered necessary.

### Statistical Analysis

Preoperative baseline characteristics, intraoperative and postoperative parameters of AAA patients and of subgroups were summarized by descriptive statistics. For continuous variables with symmetric distribution, mean data were expressed as mean ± standard deviations (SD), whereas median and interquartile range (IQR) was used for those with asymmetric distribution. Categorical variables were reported as number of patients with proportions. Fisher’s exact test was used in univariate analyses to investigate differences in the preoperative demographics and the major complications observed between those in the elective and emergency groups. Kaplan-Meier analysis and the log-rank or Wilcoxon tests were used to compare survival between both different postoperative major complications and the type of repair. A *P*-value of <.05 from two-sided tests was considered statistically significant. Analysis was performed using the R software package (http://www.R-project.org/).

## Results

A total of 1017 AAA patients diagnosed as having AAA and who had undergone open repair during the study period. Among this study population, 37 patients were excluded from the study due to missing data (n = 17), mycotic aneurysm (n = 6) and secondary OR with explant of EVAR (n = 14) leaving a total of 980 AAA for analysis. Of 980 AAA, 672 were elective OR, 308 were emergency OR (for ruptured or symptomatic). Table 1. During the same period our institution performed 1005 Endovascular aneurysm repairs (EVAR).

**Table 1.** Number of Aortic Repairs Per Year Stratified by Type of Repair.

Year	Overall	Elective	Emergency
2004	4	4	0
2005	1	—	1
2006	4	4	
2007	42	31 (+1 explant)	10
2008	50	28	22
2009	70	56 (+1 explant)	13
2010	41	22 (+4 explant)	15
2011	67	35	32
2012	88	59	29
2013	113	76	37
2014	102	70 (+4 explant)	28
2015	94	65 (+1 explant)	28
2016	88	62 (+2 explant)	24
2017	90	66	24
2018	87	55	32
2019	53 (last 3/9/19)	39 (+1 explant)	13
Total	994	672 (+14 explant)	308

Analysis of outcomes in 980 patients who underwent open repair for AAA, details of preoperative baseline characteristics, intraoperative and postoperative parameters were shown in Table 2.

**Table 2.** Preoperative Demographics.

Variable	Elective (N = 672)	Emergency (N = 308)	*P* =
Age (years)	73.3	76.1	<.0001
Gender, % Male	561/672 (83.5%)	235/308 (76.3%)	.0083
Hypertension	394/672 (58.6%)	154/308 (50.0%)	.0126
Diabetes Mellitus	77/672 (11.5%)	33/307 (10.7%)	.8275
IHD	256/671 (38.2%)	100/307 (32.6%)	.0997
CHF	20/672 (3.0%)	13/308 (4.2%)	.3410
Chronic renal disease	70/671 (10.4%)	31/308 (10.1%)	.9103
Current Smoker	199/626 (31.8%)	89/261 (34.1%)	.7016
Chronic Lung disease	120/671 (17.9%)	76/308 (24.7%)	.0160
PAD	18/671 (2.7%)	1/307 (.3%)	.0111

IHD, Ischemic heart disease; CHF, congestive heart failure; CRD, chronic renal disease; DM, diabetes mellitus; HTN, hypertension; LE, lower extremity; PAD, peripheral artery disease. Categorical variables are presented as percentage.

The mean age at repair was 73.6 ± 7.8. Median aneurysm size at the time of repair was 61 mm in the open repair Elective AAA group (IQR, 12 mm) and 78 mm in the Emergency RAAA group (IQR, 25 mm). Patients in the elective group were significantly younger (*P* = .0001), with a higher proportion of male patients (*P* = .0083) and hypertension (*P* = .01). Baseline patient characteristics are outlined in below in Table 2. In the elective group ASA class was I (.75%), II (20.4%), III (74.2%), IV (4.6%), V (0%). In the emergency group ASA class was I (.72%), II (4%), III (12.3%), IV (51.8%), V (31.2%). There were significantly more patients with ASA class IV and V in the emergency group (n = 229) than in the elective group (n = 31), *P* < .0001.

### Length of Stay (LOS), Discharge Destination, and Readmission

Median length of stay overall in hospital stay was 10 ± 19.6 days, for elective repair it was 9 days and emergency repair was 15 days. For elective repair 36.6% of patients returned to the ward post-operatively, 49% High Dependency Unit (HDU), 14.2% Intensive Care Unit (ICU), 1 patient died in theatre. For emergency repair 4.6% of patients died in theatre, 13.9% were transferred to HDU, 78.9% ICU and 2.6% returned to the ward. The median LOS in critical care for elective repair was 1 days (range 0-71 days) and for emergency repair was 4 days (range 0-70 days).

### 30-day Mortality

Overall 30 day mortality was 11.3%, elective 30 day mortality was 2.5%, and emergency 30 day mortality was 29.9%.

### Major Complications

The incidence of in-hospital Major Complications following open repair AAA diagnosed by surgeons at discharge was 38.4%. Overall major complication rate in the elective group was 29.8% and in the emergency group 56.2%. In the elective group 10.1% of patients had a post-operative cardiac complication (arrhythmia/ischemic event), 18% respiratory, 7.6% renal failure 1.6% postoperative confusion. Within the emergency group 19% had a cardiac complication, 35% respiratory, 25% renal failure, 3% post-operative confusion, Table 3.

**Table 3.** Major Complications.

Complication	Elective (%)	Emergency (%)	*P* =
Cardiac	10	19	.0003
Respiratory	18	35	<.0001
Renal failure	8	25	<.0001

Log-rank or Wilcoxon Test* analysis was carried out for in-hospital mortality of patients was presented in Table 4. Univariate analysis revealed that preoperatively Hypertension, Chronic Lung Disease, Chronic Heart Failure and Chronic Renal Disease were not significantly associated with increased mortality Table 5.

**Table 4.** Effect of Type of Open Repair, Major Complication and 30-day Mortality.

Variable	*P*-value
Sex	.0009
Type of repair*	<.0001
Type of repair (excluding explant)*	<.0001
Rupture	<.0001
Previous aortic intervention*	.109
ASA Category	<.0001
Hypertension	.0683
Chronic Lung disease	.0533
Chronic heart failure	.0189
Chronic renal disease	.1460
Stroke (pre-operatively)*	.4969
Smoking status	.4415

**Table 5.** In-Hospital Reinterventions by Cause (%).

Reintervention	Elective (%)	Emergency (%)
Embolectomy	8/47 (17%)	10/49 (20%)
Lower limb bypass	8/47 (17%)	5/49 (10%)
Explant during admission	0 (0%)	2/49 (4%)
Amputation (TT)	0 (0%)	2/49 (4%)
Amputation (TF)	1/47 (2%)	6/49 (12%)
Haemorrhage control	11/47 (23%)	12/49 (24%)
Colectomy	12/47 (26%)	14/29 (29%)
Wound Dehiscence	3/47 (6%)	2/49 (4%)
Splenectomy	2/47 (4%)	2/49 (4%)
Hernia repair	1/47 (2%)	1/49 (2%)
Graft Occlusion	1/47 (2%)	0 (0%)
Negative laparotomy	2/47 (4%)	0 (0%)

Transfemoral (TF) Amputation; Transtibial (TT) Amputation.

### Type of Open Repair and Survival

**Figure 1.**
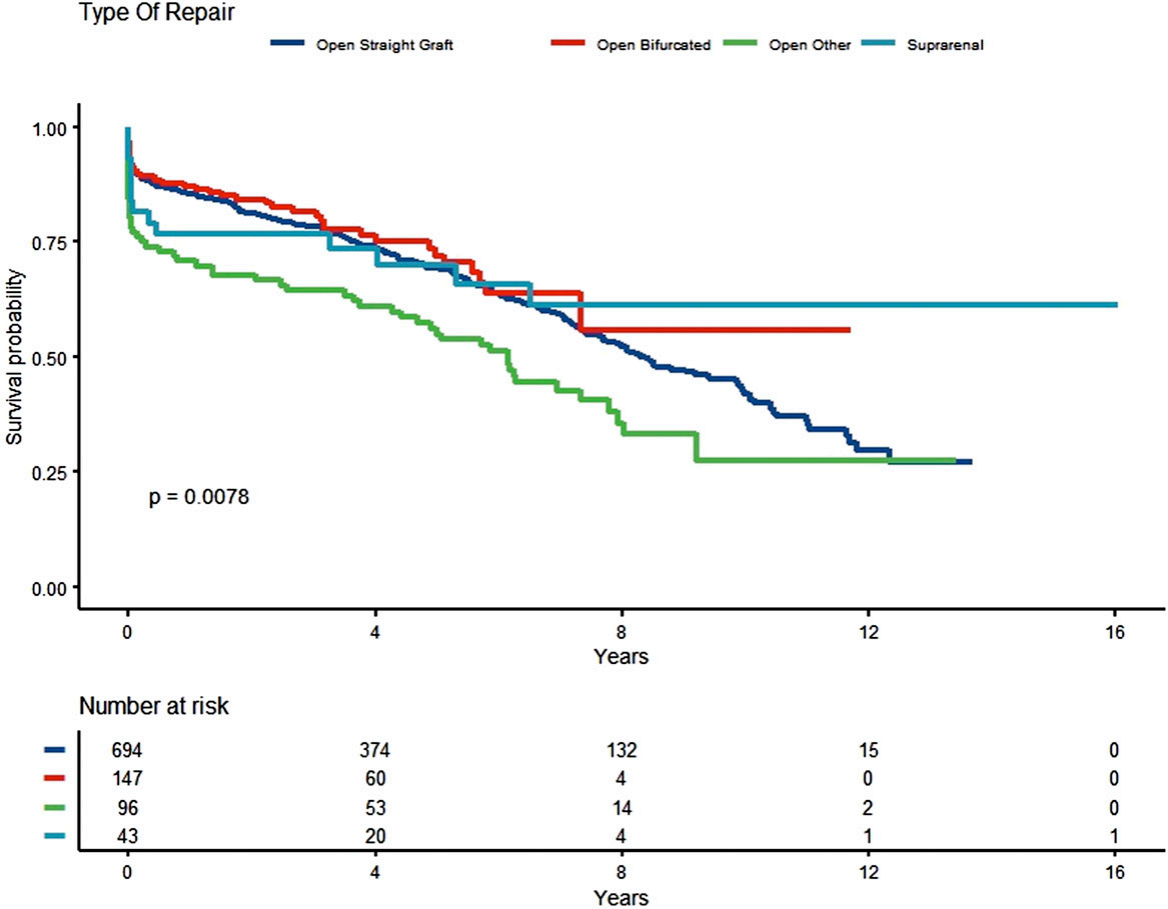
Type of open repair and survival.

In all cases a transabdominal approach was performed. Within the elective group 73% were straight graft, 17% bifurcated graft, 5% open other and 4% involved a suprarenal clamp. Within the emergency group 65% were straight grafts, 19% open other, 10% open bifurcated and 6% involved a suprarenal clamp. We found no significant difference between different types of OR (*P* = .7) and survival for either elective or emergency OR AAA (*P* = .3), including those that required a supra-renal clamp (*P* = .84), this is demonstrated in Figure 1.

### Reinterventions After Open AAA Repair

Overall, 30-day reintervention rate was (9.7%), There was a 2-fold increase in reintervention in the emergency group (15.9%) compared to the elective group (7%). The major reasons for reintervention in the elective group were haemorrhage control (23%), bowel ischaemia requiring colectomy (25.5%) and limb ischaemia requiring bypass 17% and embolectomy 17%. The major reasons for reintervention in the emergency group were lower limb ischaemia requiring bypass (10.2%) and embolectomy (20.4%) bowel ischaemia requiring colectomy (28.6%) and haemorrhage control (24.5%). Reasons for intervention are outlined in full in Table 4.

### Long-Term Survival

**Figure 2.**
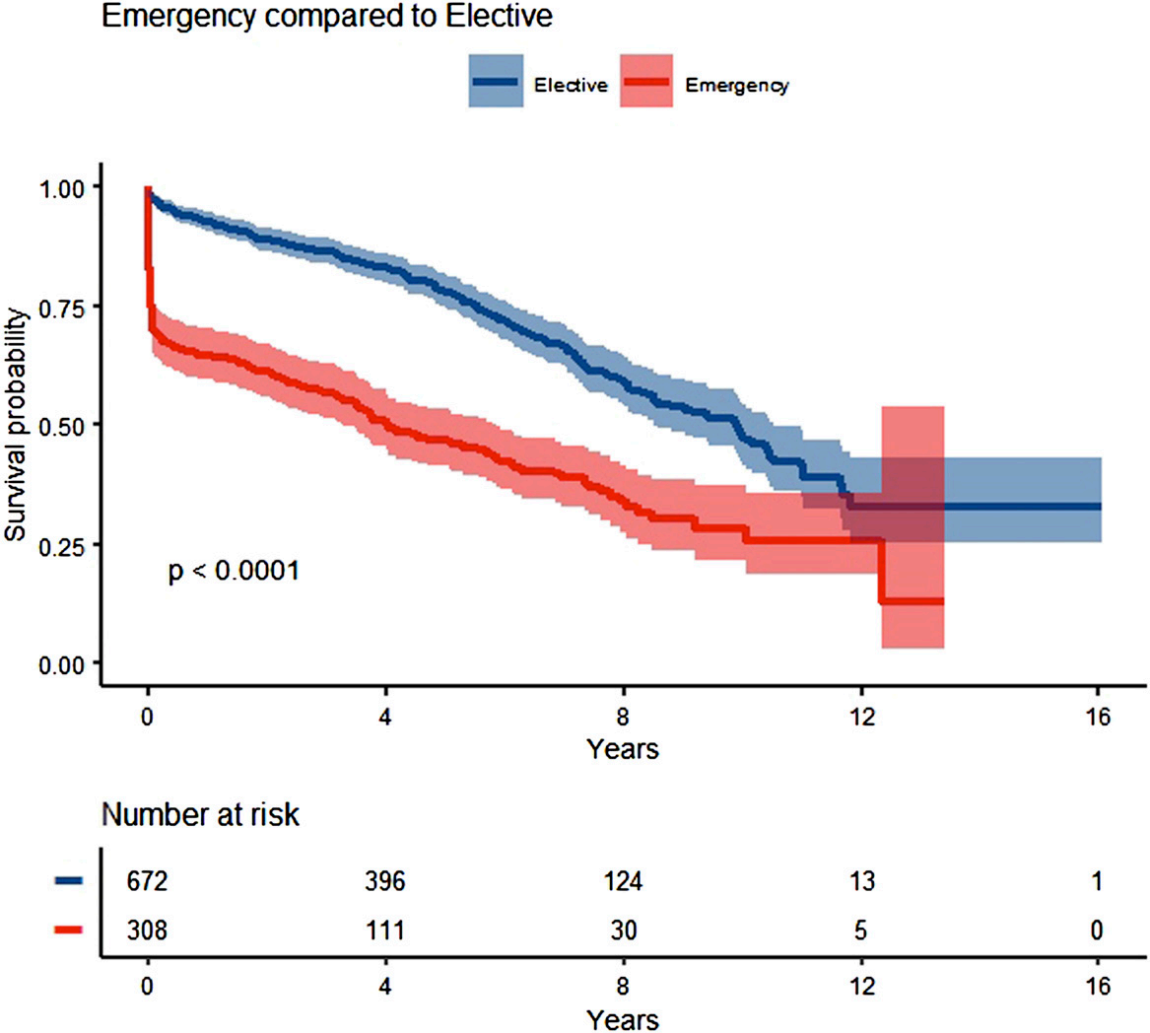
Survival after Elective v Emergency repair.

Following the initial drop off after emergency repair both elective and urgent repairs followed a similar trajectory. Figure 2 demonstrates a Kaplan-Meier survival analysis showing that the overall survival rate is significantly different for the elective group (67.6%) and emergency group (31.0%) (log-rank *P* < .0001). There was significant difference in survival at 1 year (*P* < .0001), 5 years (*P* < .0001) and 10 years (*P* = .0002) between elective and emergency groups, Figure 2.

## Discussion

This study is 1 of the largest cohorts to examine short and long-term outcomes after elective and emergency open repair (OR) of abdominal aortic aneurysm (AAA). We considered 1017 consecutive patients and after exclusions report the outcomes of 980 consecutive patients treated by OR, 672 elective and 308 emergency (for rupture or symptomatic), between 2004 and 2019 in the largest AAA treatment centre in the UK.

We report an overall 30 day mortality rate of 11.3%, elective 2.5%, and emergency 29.9%. These mortality rates are in accordance with historical cohorts^8^ and the OR outcomes in the major randomised controlled trials (RCT) for elective repair of AAA and emergency repair of rupture abdominal aortic aneurysm (RAAA).^12-14^ Early mortality for elective OR, is higher than comparable contemporary cohorts undergoing endovascular AAA repair (EVAR), both in our institution and by comparison with peers, and reflective of RCTs.^11,12^ However, that must be balanced against recent concerns regarding the increased re-intervention rates and late aneurysm-related mortality after EVAR.^6,15^ The mean age at repair overall was 73.6 years, with elective patients on average 6 years younger than emergency patients. The age range was (35 to 94) and the distribution of older patients in the elective group included over 80 s (n = 99) and over 90 s (n = 0) older age was significantly associated with mortality in our series (*P* < .0001). However despite this our results correspond with several studies which have shown elective OR can be safely carried out even in selected octogenarians and nonagenarians.^16-18^ Female patients were significantly more likely to present as an emergency (*P* = .008), which may be reflective of the success of the current screening paradigm in the United Kingdom, with men in their 65^th^ year offered a one-off ultrasound scan. Our median aneurysm size at the time of repair was 61 mm in the OR Elective AAA group (IQR, 12 mm) and 78 mm in the Emergency OR group (IQR, 25 mm).

Patients with renal impairment had significantly higher mortality rates after both elective (*P* = .019) and emergency (*P*= .02) repair differing from a previous study showing that patients with renal impairment had no difference in mortality after elective OR but is associated with increased mortality after emergency repair,^19^ whereas end-stage renal disease or pre-operative dialysis does predict high mortality in both elective and emergency OR and even survivors have limited life-expectancy after OR.^20^

The overall, 30-day reintervention rate was (9.7%), in the elective group (7.0%) and emergency group (15.9%). There was a 2-fold increase in reintervention in the emergency group compared to the elective group. The major reasons for reintervention in the elective group were limb ischemia (34%), haemorrhage control (23%) and bowel ischaemia requiring colectomy (25.5%). The major reasons for reintervention in the emergency group were lower limb ischaemia (30%), bowel ischaemia requiring colectomy (28.6%) and haemorrhage control (24%). Limb ischaemia was the commonest reason for reintervention overall and in the elective group an attempt at limb salvage by revascularisation was made in all cases, with 16 limbs undergoing femoral embolectomies and 8 limbs undergoing femoro-popliteal bypass using above knee synthetic grafts in all cases, and failure of revascularisation requiring transfemoral amputation occurred in only 2 cases. Limb ischaemia was again the commonest reason for reintervention in the emergency group, where it was twice as common as in the elective group, and whilst revascularisation was attempted in 15 patients, with 10 embolectomies and 5 femoro-popliteal bypasses, there was a higher failure rate with 9 amputations (2 transfemoral and 7 transtibial). Another important complication noted is that of colonic ischaemia requiring emergency laparotomy and colectomy, with 12/672 (1.8%) in the elective group and 14/308 (4.5%) in the emergency group. Colectomies were more than twice as common after emergency OR and this may be expected given the high ASA Class of this cohort and the presence of haemodynamic instability and hypotension. Our results are comparable with other series which confirm reinterventions are common after OR AAA, especially after emergency OR.^21,22^ Colonic ischaemia can be particularly difficult to diagnose and even after emergency colectomy the risk of mortality is high.^23,24^

The median length of stay overall in hospital stay was 10 days, for elective repair 9 days and emergency repair 15 days. The level of care required for immediate post-operative care in elective patients was a specialist vascular ward for one-third, HDU for one-half, and only 14.2% needing admission to the ICU. This practice of selective use of ICU for elective OR AAA has also recently been shown to be safe and cost effective.^25^ Emergency OR were invariably recovered in HDU or ICU. When considering survival after discharge there remained a significant difference between the elective at 1 (*P* = .03) and 5 (*P* = .0002) years, whereas over the long-term those who survived for 10 years there was no significant difference (*P* = .14). However, it is reassuring that long-term survival after elective and emergency OR AAA is excellent and in keeping with other studies.^5,26^

### Limitations of this Study

This study has some limitations. This was a retrospective study of prospectively collected data of a non-randomized design. Retrospective studies lack exposure control or the reporting on some variables and introduce selection and misclassification bias. However, the data in our study were collected in a prospective robust manner that reduces selection and information bias. It is of note that over 2000 AAA were treated at our institution over the study period with roughly half treated by OR and half by EVAR and has remained relatively consistent annually over the duration of this study. Our institution has noted the ebb and flow of opinion in respect to AAA treatment of choice and has taken a consistent selective approach to consideration of preferred treatment, by either OR or EVAR, which is team-based and patient-centred considering patient-related factors and choice. If any criticism could be accepted here it would be that our OR cohort has had more challenging anatomy than most other large series and RCT.

## Conclusions

Our data confirm that open surgery for AAA can be performed in large volume centres quite safely and with results comparable to the major Randomised Clinical Trials (RCTs). Elective and Emergency surgery does affect early 30 day mortality but does not influence long-term outcomes. Open surgery should still be considered the standard in the management of AAA.
